# Editorial: Heart Rate Variability and Other Autonomic Markers in Children and Adolescents

**DOI:** 10.3389/fphys.2019.01265

**Published:** 2019-10-11

**Authors:** George E. Billman, Jerzy Sacha, Bozena Werner, Piotr Jerzy Jelen, Jakub S. Gąsior

**Affiliations:** ^1^Department of Physiology and Cell Biology, The Ohio State University, Columbus, OH, United States; ^2^Faculty of Physical Education and Physiotherapy, Opole University of Technology, Opole, Poland; ^3^Department of Cardiology, University of Opole, Opole, Poland; ^4^Department of Pediatric Cardiology and General Pediatrics, Medical University of Warsaw, Warsaw, Poland; ^5^Department of Biophysics and Human Physiology, Medical University of Warsaw, Warsaw, Poland; ^6^Faculty of Health Sciences and Physical Education, Kazimierz Pulaski University of Technology and Humanities, Radom, Poland; ^7^Clinical Department of Cardiology at Bielanski Hospital, Institute of Cardiology, Warsaw, Poland

**Keywords:** heart rate, heart rate variability, autonomic nervous system, infants, children, adolescents

Despite thousands of articles addressing heart rate variability (HRV) in healthy subjects and patients with various clinical conditions published during the last decades (Billman, 2011), our understanding of the development of the cardiac autonomic nervous system is still very limited. During maturation, from infancy through adolescence to adulthood, changes in cardiac autonomic neural regulation elicit corresponding changes in cardiac rhythm, changing both heart rate (HR) and its beat-to-beat variability. These fluctuations in heart rate display marked regularity, corresponding with changes in respiration (i.e. HR increases during inspiration and decreases with expiration), and are thought to reflect changes in a cardiac autonomic regulation (Billman, 2011). For example, it is widely accepted that the high frequency component of the R-R interval variability is dominated by changes in cardiac parasympathetic efferent nerve activity while the relationship, if any, between cardiac autonomic regulation and the low frequency component of this variability is much more complex and the subject of considerable debate (Houle and Billman, 1999; Billman, 2011, 2013a). Despite this controversy, various mathematical approaches have been developed, both to investigate cardiac autonomic regulation in healthy individuals and to identify changes that might be associated with an increased risk for adverse cardiac events (Billman, 2011). However, the majority of these studies have focused on changes in adult populations. Furthermore, it is not widely appreciated that, as a consequence of the non-linear inverse relationship between HR and R-R interval, similar changes in HR can provoke profoundly different values for HRV (i.e. the R-R interval variability) depending on the prevailing average HR: bigger values for HRV at a lower prevailing HR than at a higher HR (Sacha and Pluta, 2005; Billman, 2013b; Sacha, 2014a,b). Consequently, any changes in HR usually entail simultaneous changes in HRV.

The most common developmental phenomenon is a reduction of HR with a child's age, which in turn, may influence HRV. The extent to which alterations of HRV during growth and development result from this HR change remains to be determined. The normalization of HRV for the prevailing HR liberates HRV from the influence of HR and thereby allows for the objective assessment of the cardiac autonomic influences on this variability, separating mathematical from physiological changes in HRV (Sacha and Pluta, 2005; Billman, 2013b; Sacha, 2014a,b). Moreover, other autonomic markers such as deceleration capacity and indices of baroreceptor reflex sensitivity are also sensitive to changes in prevailing HR and could be effected by developmental changes in HR. It is therefore critical to assess both tonic and reflex autonomic markers independently of average HR in order to evaluate changes in cardiac autonomic regulation during maturation from the infant to the young adult.

As previously noted, there are both linear and non-linear components to HRV, yet the effects of development on non-linear heart rate dynamics remain to be determined. Although a number of non-linear autonomic markers have been proposed and tested in adult populations (Billman, 2011), little is known about either the effects of maturation on these markers or the effects of changes in prevailing HR during growth and development. It is also unclear whether cardiac autonomic function becomes more linear or non-linear with age in healthy children. Finally, the relationship between changes in indices of HRV during the maturation process and abnormalities/diseases in pediatric populations also remains to be determined. In other words, can changes in HR and its variability be used as part of the differential diagnosis process to identify specific pathologies and those individuals at the greatest risk for complications from disease, as has been proposed for adult populations (Billman, 2011)? It is the purpose of this monograph to provide a comprehensive assessment of developmental changes in both HR and HRV in infants, young children, and adolescents. Particular emphasis is placed on the contribution of HR to alterations in HRV during maturation in healthy populations. In addition, HRV indices are evaluated as potential markers for the identification of individuals at risk for pediatric diseases. The book is divided into the three main section: HRV in infants and young children (<3 years of age), young children (3–12 years of age) and adolescents to young adults (13–21 years of age). A brief summary of the chapters contained in each section follows.

The first section examines HRV in infants and young children. In chapter 2, Oliveira et al. evaluate changes in HRV during the first 24 h after birth, reporting that HRV increases during the first 6 h and then declines toward new steady state values. They further find a strong correlation between reduced HRV and clinical risk factors, concluding that HRV is a good indicator of overall well-being and can be used as a marker of birth-related stress. Chapter 3 compares the impact of paternal or maternal stroking touch on 4–16 week old infants, reporting that touch by either parent increased respiratory sinus arrhythmia amplitude (Van Puyvelde et al.). Chapter 4 closes this section, with an investigation of HRV for risk assessment in toddlers (18–36 months old) with and without developmental difficulties (i.e., social dysfunction). Billeci et al. report that the HRV response to a joint attention task (defined as the ability to coordinate visual attention with another person and then shift the gaze toward an object or event) was attenuated in toddlers exhibiting signs of social dysfunction (autism spectrum disorders) compared to typically developed children.

The second section, investigates HRV in young and pre-adolescent children. Chapter 5, Gąsior et al. examine a number of HRV indices in children ages 6–13 years (non-athletes) in order to establish the normal range of these values after correction for prevailing HR. Their study provides important normative values for these various HRV indices, finding that they vary independently of either gender or changes in HR. This chapter also provide a link to supplementary material that contains mathematical tools that can be used to correct HRV for HR. In a similar manner (chapter 6, Bobkowski et al.), investigate the association between age and gender on non-linear indices of HRV in young children and adolescents (ages 3–18 years). They report that these non-linear indices were not affected by gender but increased with age. Chapter 7–10 then evaluate the association between body composition and physical activity on HRV in children. For example, Herzig et al. (chapter 7), assess the association between physical activity and/or body mass on HRV after correction for age-related changes in HR in children aged 2–6 years. They report that HR and skin fold thickness both decrease with increased levels of habitual physical activity. Importantly, indices of HRV increased with age and activity only after adjustment for age-associated reductions in prevailing HR. In chapters 8 and 9, Plaza-Florido et al. further characterize the effects of HR on HRV in overweight and clinically obese children. They report that HRV declines with increasing weight. However, this weight-associated decline in HRV is eliminated by correction for prevailing HR (chapter 8, Plaza-Florido et al.). They (chapter 9, Plaza-Florido et al.) further demonstrate that physical activity reduces HR and increases HRV but, once again, the increase in HRV is eliminated by adjustment for HR. These results further emphasize that HRV indices must be adjusted for prevailing HR in order to reveal true differences or changes in cardiac autonomic regulation. The relationship between the energy-related biomarkers, leptin and adiponectin, and HRV in boys and girls is examined in chapter 10. Specifically, leptin was a negative predictor of “parasympathetic” regulation in boys and was associated with higher values of low frequency power in girls (Van De Wielle and Michels). In contrast, adiponectin was a negative predictor of HRV in girls but not in boys after adjustment for prevailing HR (Van De Wielle and Michels). Chapter 11 investigates the relationship between non-linear indices of HRV and psychological disorders in children aged 9–13 years. Fiskum et al. report that non-linear indices of HRV were inversely related to the severity of the psychopathology: higher values of HRV were associated with lower incidence of disorder. Similarly, children (mean age 8 years old) with developmental coordination disorders exhibit lower HRV in the supine position and a blunted response to an orthostatic challenge than did children without coordination disorders (chapter 12, Cavalcante -Neto et al.).

The final section examines HRV variability in adolescents and young adults. The results of a meta-analysis that included about 5,000 children aged 12–17 years are presented in chapter 13. In contrast to studies that include younger children, girls have higher prevailing HRs and lower HRV than boys (Koenig et al.). Two different approaches to reduce the effects of HR and HRV, using computer stimulation studies on data obtained from males aged 19–38 years, are compared in chapter 14. Both regression analysis formulae and interpolating an R-R interval time series effectively attenuated the effects of HR on HRV (Bolea et al.). This section closes with an examination of the effects of physical activity (chapter 15) on HR and HRV as well as an investigation of the relationship between HRV and internalizing disorders (depression and anxiety) (chapter 16) or sleep stability (chapter 17). In a cross-sectional study of young athletes aged 10–19 years (Subramanian et al.), exercise is associated with lower baseline HR and increased indices of cardiac parasympathetic regulation, just like in younger children. Conversely, anxiety and depression were more common in adolescent girls than in similarly aged boys, and these psychological disorders were accompanied by lower HRV and cardiac complexity (Fiol-Veny et al.). The authors suggest that these indices may help identify individuals particularly sensitive to psychological stressors. Finally, Cysarz et al. investigate the association between aging related changes in sleep patterns (duration and stability) and HRV as measured by cardiopulmonary coupling analysis (CPC, the degree of coherent coupling between HRV and variations in the amplitude of the R wave produced by changes in tidal volume that occur during respiration) in children and young adults (ages 4–22 years). They find that both sleep stability and CPC decline with age.

The authors hope that this brief monograph will serve as a guide to how to use linear and non-linear indices of HRV appropriately (i.e., after adjustment for age-related changes in HR). Both research investigators and clinicians will then be able to use HRV indices not only to evaluate developmental changes in cardiac regulation but also to aid in the identification and treatment of individuals at risk for pediatric diseases.

## Author Contributions

The editorial was written by GB and proof read and approved by the co-authors for submission.

### Conflict of Interest

The authors declare that the research was conducted in the absence of any commercial or financial relationships that could be construed as a potential conflict of interest.
